# Cheese Whey Processing: Integrated Biorefinery Concepts and Emerging Food Applications

**DOI:** 10.3390/foods8080347

**Published:** 2019-08-15

**Authors:** Iliada K. Lappa, Aikaterini Papadaki, Vasiliki Kachrimanidou, Antonia Terpou, Dionysios Koulougliotis, Effimia Eriotou, Nikolaos Kopsahelis

**Affiliations:** 1 Department of Food Science and Technology, Ionian University, Argostoli, 28100 Kefalonia, Greece; 2 Department of Food and Nutritional Sciences, University of Reading, Berkshire RG6 6AP, UK; 3 Department of Environment, Ionian University, Panagoula, 29100 Zakynthos, Greece

**Keywords:** food processing, integrated biorefineries, circular-economy, whey proteins, lactose esters, prebiotics, hydrogels, edible films, bacterial cellulose, carotenoids

## Abstract

Cheese whey constitutes one of the most polluting by-products of the food industry, due to its high organic load. Thus, in order to mitigate the environmental concerns, a large number of valorization approaches have been reported; mainly targeting the recovery of whey proteins and whey lactose from cheese whey for further exploitation as renewable resources. Most studies are predominantly focused on the separate implementation, either of whey protein or lactose, to configure processes that will formulate value-added products. Likewise, approaches for cheese whey valorization, so far, do not exploit the full potential of cheese whey, particularly with respect to food applications. Nonetheless, within the concept of integrated biorefinery design and the transition to circular economy, it is imperative to develop consolidated bioprocesses that will foster a holistic exploitation of cheese whey. Therefore, the aim of this article is to elaborate on the recent advances regarding the conversion of whey to high value-added products, focusing on food applications. Moreover, novel integrated biorefining concepts are proposed, to inaugurate the complete exploitation of cheese whey to formulate novel products with diversified end applications. Within the context of circular economy, it is envisaged that high value-added products will be reintroduced in the food supply chain, thereby enhancing sustainability and creating “zero waste” processes.

## 1. Introduction

Design of integrated biorefinery concepts endeavors a consolidated approach to valorize all possible waste and by-product streams under the concept of circular economy. In line with this, it is a prerequisite to target the formulation of multitude products rather than a single product to secure sustainable processes. On top of that, the high-value products will need to find value-added end applications, whereas the food sector is deemed of high importance. Food products with added value will ultimately meet consumers’ demands and confer possible health benefits.

Cheese whey constitutes a by-product of the dairy industry and refers to the liquid stream deriving from the transformation of milk into cheese, specifically from the process of agglomeration of casein micelles. Whey is mainly composed of water, but also contains around 50% of the milk solids [1,2]. The dry matter fraction retains most of the lactose (66–77%, *w*/*w*), 8–15% (*w*/*w*) of numerous types of globular proteins, along with 7–15% (*w*/*w*) of minerals salts [3]. The amount of whey generated relates to the amount of cheese production and also to the productivity based on the type of milk, whereby approximately 9 L of whey are obtained for every 1 kg of cheese produced [4,5].

Over the last decades, cheese whey is considered the most important pollutant of the dairy industry, associated with serious environmental hazards in the case that designated sustainable treatments are not applied. The major issue lies in the high organic load, mainly due to the high content of lactose but also to the occurrence of hardly-biodegradable proteins [6]. More specifically, chemical oxygen demand (COD) of cheese whey can vary from 50,000 to 80,000 mg/L, whereas biochemical oxygen demand (BOD) is around 40,000 to 60,000 mg/L [7]. The rapid consumption of oxygen in the soil caused from the breakdown of proteins and sugars present in whey poses a significant disposal problem, in line with the vast amounts of volumes generated. The global whey production in 2016 was estimated at 200 million t with an annual linear increase of 3% for the last 21 years [8].

Nonetheless, in the last decades, cheese whey characterization has been altered from waste to dairy side-stream product. Significant research has been conducted to mitigate viable and environmentally benign valorization alternatives for cheese whey, rather than just field disposal [9,10]. The high nutritional value of cheese whey [11,12] has induced the valorization of approximately 50% of residual whey [13,14,15] towards the generation of value-added products for food and chemical industries. Traditional uses of whey protein as a health promoter have been earlier reported, both in human and animal nutrition [16]. Furthermore, several process technologies and biotechnological approaches have also been developed to convert this by-product into a resource of valuable components or into an ample range of marketable beverages [17,18,19]. Scientific studies have demonstrated the nutritional and functional value of whey protein and have focused on developing a number of recovery methods via physicochemical processes [20,21,22]. Advanced technologies such as ultrafiltration and nanofiltration have enhanced the exploitation of whey streams [5,23,24]. Besides the implementation of these techniques, the deproteinized cheese whey constitutes a lactose-rich fraction, and still displays a BOD_5_ >30 kg m^−3^ [25]. Equally, the obtained fraction exhibits a high organic pollutant that should be further treated or employed as onset material for valorization processes.

Microbial-based processes to convert cheese whey into valuable products have flourished as a potential route for biorefinery development. Fermentation processes could significantly decrease the organic load (lactose content), thus enabling an economical and feasible alternative utilization of cheese whey, thereby reducing the environmental impact. However, to the best of our knowledge, there are scarce studies performed to evaluate both whey fractions in the frame of an integrated and consolidated approach which could find applications within the food industry itself. Bioprocess integration is defined as the simultaneous incorporation of more than two-unit operations in a single process, thereby enabling the utilization of an organic-rich effluent like cheese whey to generate multiple additional products.

The purpose of this study is to elaborate an overview on the conversion of whey deriving from cheese manufacture to high value-added products. Recent developments and new insights in the processing and refining technologies for cheese whey exploitation are reported, while advanced approaches with special focus on food applications are taken into consideration. The overall aim of this article is to explore potential schemes that could be applied for this by-product, by introducing the concept of novel biorefineries. Novel and cost-effective exploitation concepts have emerged to be of paramount importance, thus potential research gaps are also identified by proposing holistic approaches of cheese whey valorization to formulate a multitude of end-products. Therefore, biorefining processes that implement the valorization of lactose and whey protein towards the formulation of high value-added products through enzymatic, microbial, and chemical methods are proposed. Ultimately, it is anticipated that novel and functional foods with enhanced properties will be the target end-products, allowing the reintroduction in the food manufacturing sector, within the concept of transitioning to a closed-loop circular economy.

## 2. Bioprocess Development Using Whey Lactose

The deployment of cascade bioprocesses, to foster a holistic approach for cheese whey valorization and mitigate its disposal, has gained significant scientific attention during the last years. Pharmaceutical and food industries exhibit potential market outlets for the lactose fraction deriving from cheese whey. Special attention is given in the production of added-value compounds as a result of enzymatic catalysis or microbial fermentations. In this context, the production of lactic acid, ethanol, microbial lipids, microbial biomass, single cell protein, poly-hydroxyalkanoates, enzymes, and endo-polysaccharides has been addressed in numerous studies dealing with the exploitation of whey lactose [13,26,27]. However, this article will focus on novel, promising, and not fully developed or extensively studied bioprocesses from whey lactose, targeting food applications in the context of functional food manufacture. In particular, the synthesis of lactose derivatives, mainly as novel and targeted prebiotic oligosaccharides and fatty acids esters, has lately attracted great interest [28,29,30]. Likewise, various food additives and functional components, such as biocolorants, medicinal mushrooms, spirulina, etc., can be produced through microbial fermentations. These aspects and other recent trends in the field of whey lactose upgrading are described in the following sections.

### 2.1. Enzymatic Bioprocesses

#### 2.1.1. Galacto-Oligosaccharides

The prebiotic definition has been constantly evolving since the first definition in 1995 by Gibson and Roberfroid [31], being recently revised as “a substrate that is selectively utilized by host microorganisms conferring a health benefit” [32]. Prebiotic oligosaccharides are non-digestible compounds, varying in the composition and configuration of monosaccharide residues and the type of glycosidic linkages. Prebiotics confer beneficial effects on human health, primarily by modifying the indigenous colonic gut microbiota [33]. Prebiotic oligosaccharides can be found in fruit, vegetable, dairy, and seafood processing by-products, while they can also be enzymatically synthesized [34]. Galacto-oligosaccharides (GOS) and lactulose are well established and recognized prebiotics, based on their health-promoting effects, including immunomodulation, lipid metabolism, mineral absorption, weight management, and obesity-related issues, among others [35].

Galacto-oligosaccharides are non-digestible, galactose-containing oligosaccharides with a unit of terminal glucose in the form Glu α1–4(β Gal 1–6)n, showing a degree of polymerization (DP) ranging from 2 to 8–9 [36,37]. GOS are produced by lactose, through the transgalactosylation action of β-galactosidase (EC 3.2.1.23), yielding a mixture of oligosaccharides, mono- and disaccharides, with a high range of linkages, mainly β1–4 and β1–6, but also β1–3 and β1–2 [38]. GOS occur naturally in the milk of animals and humans at low concentrations, but they are mainly produced by chemical glycosylation or enzymatic routes to meet market demands. The worldwide market size of GOS was estimated at $703.8 million in 2017, and it is anticipated to increase significantly by 2025, following the constantly-rising demand for the consumption of dietary supplements [39]. The prebiotic effect of GOS has been widely demonstrated during in vitro animal and human studies (including studies with infants). GOS have been recognized as safe (GRAS) in the United States and are characterized as foods for specific health use (FOSHU) in Japan, where they have been applied in a spectrum of end-products such as sweeteners, bulking agents, and sugar substitutes [38,40,41]. Thereby, GOS are mostly applied to infant formula products, aiming to formulate products similar to human milk composition, but also in beverages, meal replacers, flavored milk, and confectionery products (e.g., bread). Their incorporation into food products has been regulated in many countries, which are using GOS as functional food ingredients, whereas in Europe, they are under pre-screening evaluation from the European Food Safety Authority (EFSA) [41,42]. Apart from the food industry, GOS have found applications in the animal feed, cosmetic, and pharmaceutical industries.

Another lactose-derived prebiotic is lactulose (4-*O*-β-d-galactopyranosyl-d-fructose), a non-digestible synthetic disaccharide, comprising of fructose and galactose [30]. Lactulose has been marketed mainly as a medical product [37], finding many applications in food products such as milk for bottle-fed babies to adjust the composition of their colonic microbiota [43].

The chemical route is a common method for the production of GOS and lactulose. The main drawbacks of the chemical synthesis are the requirement of catalysts and chemicals, the low specificity, and the production of undesirable compounds. However, many companies have employed enzymatic synthesis, as it offers several advantages, including the requirement of non-purified substrates, selectivity, mild reaction conditions, and lower downstream operation costs [30]. The enzymatic production and the final configuration of GOS, in terms of molecular weight distribution and linkages, are affected by various factors, including the concentration of lactose and water during the reaction and the source of the enzyme employed. The biocatalyst β-galactosidase can be obtained from several microbial sources, including *Kluyveromyces lactis*, *Bacillus circulans*, *Bifidobacterium bifidum*, *Aspergillus oryzae,* and *Streptococcus thermophiles* [44]. During GOS synthesis with β-galactosidases, lactose acts both as donor and acceptor of the transgalactosylated galactose [45], whereas during lactulose synthesis, lactose is the galactosyl donor and fructose acts as the acceptor. However, a mixture of lactulose and GOS is produced during lactulose synthesis, as lactose and fructose are simultaneously present in the reaction medium and, thus, can act as acceptors and their production ratio depends on the process conditions [46]. Likewise, GOS purification steps are essential when food applications are targeted. Hernández et al. [36] evaluated several fractionation techniques, showing the potential of yeast treatment to obtain high purity GOS, compared to diafiltration and activated charcoal [36].

The use of whey lactose has been suggested as an alternative substrate for the enzymatic synthesis of potential prebiotics, leading to a more sustainable and competitive process within the concept of bioeconomy. Although there are many studies reporting GOS production using primarily pure lactose [28], research has been also conducted using whey lactose as substrate. Lactose from whey can be obtained via crystallization of a supersaturated solution [41]. Wichienchot and Ishak [47] suggested that lactose derived from cheese whey is a potential source for GOS and lactulose production [47]. Splechtna et al. [48] found that GOS production, catalyzed by a β-galactosidase of *Lactobacillus* sp., was reduced compared to buffered lactose substrate, whereby GOS yield was 28% of total sugars. However, higher yields have been reported by other studies [48]. Das et al. [49] reported 77% GOS production from whey lactose by employing β-galactosidase from *Bacillus circulans* [49]. High GOS production (53.45 g/L) has been also produced from lactose-supplemented whey, catalyzed by the β-galactosidase of *Streptococcus thermophilus* [35]. Díez-Municio et al. [50] indicated that cheese whey is a suitable material for the synthesis of the trisaccharide 2-α-d-glucopyranosyl-lactose [50]. The authors mentioned a yield of 50% of the initial amount of lactose, under the optimum reaction conditions. A co-reaction was performed using bovine cheese whey and tofu whey as lactose and sucrose sources, respectively, for the production of 80.1 g/L lactosucrose. This approach allowed the simultaneous utilization of two by-products which resulted in a very high productivity of 40.1 g lactosucrose /L/h [51]. In another study, a continuous reaction was performed using β-glucosidase from *Kluyveromyces lactis,* achieving a maximum yield of 31% oligosaccharides in a pilot plant scale UF-hollow fiber membrane reactor [52]. Lower yields up to 11.3 % of GOS have been obtained in other studies using different types of cheese whey (sweet whey, acid whey) [53]. Overexpression of β-galactosidase from *S*. *thermophilus* in a food grade *L*. *plantarum* strain resulted in the production of 50% of GOS using 205 g/L of lactose derived from whey [54], thus indicating an efficient valorization route for whey lactose.

For the commercial production of GOS, Nestle company has developed a procedure using partially demineralized sweet whey permeate. Initially, whey is concentrated and then β-glucosidase produced from *A. oryzae* is added and the reaction is stopped through heat inactivation of the enzyme [42].

Scott et al. [55] performed a techno-economic analysis to evaluate the production of whey powder and lactose as market outlet for subsequent GOS production [55]. The plant capacity, along with the current prices of whey powder and lactose, were closely affected with the profitability of the complete process. Nonetheless, the authors suggested that the bioprocess and restructuring of the plant could become more robust if the price of whey powder rises [55]. On the other hand, the development of integrated cheese whey biorefineries towards the production of added-value products, from both lactose and whey protein streams, could exploit the full potential of cheese whey, including process optimization and downstream recovery that could be annexed to other bioprocesses. Added-value products from whey protein, and the possibility to configure cascade bioprocessing for cheese whey, will be elaborated in the following sections, proposing the development of robust integrated scenarios. 

#### 2.1.2. Lactose Fatty Acid Esters

Sugar esters are odorless, non-toxic, and biodegradable compounds of high importance for the food industry [30]. The most common sugar esters derive from sucrose, with an estimated global market of $74.6 million in 2020 [56]. Although, lactose esters have not been extensively studied, they have found several applications within the food, cosmetic, and pharmaceutical industries [57]. These sugar esters demonstrate excellent emulsifying and stability properties in food products, whereas they may be applied as low-fat alternatives. Additionally, they present antimicrobial activity against many foodborne pathogens, as well as medicinal properties such as anticancer activity [30,57].

Chemical synthesis of lactose esters is the most common route for their production. The main drawback of the chemical lactose esterification is the production of non-stereospecific esters [57]. The use of enzymes, such as lipases, esterases, and proteases, affects the reaction selectivity due to their regiospecifity [57]. Among all enzymes, lipases have attracted significant interest due to their stability during several batch reactions at high temperatures and their ability to utilize different substrates [57,58,59,60,61].

Lactose ester production has been studied since 1974 [62]. An extensive review for lactose ester production through enzymatic catalysis demonstrated that enzymes from various microbial sources—e.g., *Candida antarctica*, *Mucor miehei,* and *Pseudomonas cepasia,* among others—can be utilized, entailing high yields. More specifically, lipases have achieved yields up to 89%, whereas the protease from *Bacillus subtilis* reached the highest yield (96%), at mild temperature conditions (45 °C) [63,64]. During sugar ester synthesis, fatty acid vinyl esters are utilized as acyl donors. Since vinyl esters are expensive and result in unstable by-products (vinyl alcohols), Enayati et al. [65] replaced them with fatty acids, such as lauric acid and palmitic acid, which yielded high lactose ester synthesis (93%) [65]. This method could be further developed by employing renewable resources with a high content of free fatty acids, such as fatty acid distillates. For instance, palm fatty acid distillate has been successfully valorized towards polyol ester production using a commercial lipase [61]. Even though lactose esters have been recognized for their superior properties and as attractive substitutes of synthetic surfactants [65,66], only pure lactose has been employed for their production until now [57].

### 2.2. Microbial Bioprocesses

#### 2.2.1. Food Biocolorants and Aroma Compounds

Carotenoids are considered one of the most important groups of natural pigments, exhibiting numerous biological functions. Carotenoids are characterized by their antioxidant activity and their exceptional health benefits on human health, such as the reduction of cardiovascular diseases, anti-diabetic, anti-cancer, and anti-inflammation activities [67,68,69]. Humans are not able to synthesize carotenoids; thus, their uptake can be only performed via the consumption of carotenoid-rich food products. The most commercially important carotenoid is β-carotene, followed by lutein and astaxanthin. Likewise, β-carotene is widely applied as a food supplement, acting as provitamin A, and as a coloring agent in food products, such as butter, margarine, cheese, confectionery, ice cream, juices, other beverages, etc. [70,71]. Natural astaxanthin has gained industrial interest as it presents significantly higher antioxidant activity than the respective counterpart made via the chemical route [72]. Astaxanthin is widely utilized in salmon aquaculture and as a dietary-supplement for human consumption [71,73].

Carotenoids were initially extracted from plants, but they are currently produced primarily through chemical synthesis. Natural origin carotenoids can be obtained only through plant extraction or biotechnologically. The fermentative production of carotenoids has been well-investigated using various carbon sources, such as glucose, sucrose, and xylose, among others; however, the interest has been shifted to the use of low-cost substrates, aiming to reduce the high production cost. In this context, there is a growing interest for the development of bioprocesses using renewable resources as alternative carbon sources [71]. The fungus *Blakeslea trispora*, as well as many yeast species belonging to the genera of *Rhodosporidium* sp. *Rhodotorula* sp. and *Phaffia rhodozyma,* have been studied for carotenoid production using low-cost substrates [71,74]. Usually, most of them produce a mixture of carotenoids consisting of β-carotene, torulene, torularodine, and γ-carotene [71]. In the case of *P. rhodozyma,* the carotenoid mixture primarily comprises astaxanthin [75]. The microalgae *Haematococcus pluvialis* also constitutes a rich source of astaxanthin, thus presents the highest potential for astaxanthin production [73].

Among renewable resources, cheese whey has emerged as a promising candidate for carotenoid production, however only a few studies are found in the literature. Cheese whey, or deproteinized cheese whey, has been utilized for the production of carotenoids using various microorganisms. Nevertheless, most of them are lactose-negative species, thus in many cases, enzymatic hydrolysis of deproteinized cheese whey is carried out prior to fermentation. Table 1 summarizes all the results with respect to carotenoid production from cheese whey up-to-date. Evidently, *B. trispora* demonstrates the highest yields among all microorganisms. The highest carotene production of 1620 mg/L with an intracellular yield of 222 mg/g was reported by Roukas et al. [76]. The fermentation was carried out in a bubble column reactor using deproteinized, hydrolyzed cheese whey. This is among the highest values achieved with agro-industrial by-products, indicating that cheese whey might be one of the most promising renewable resources for the commercial production of carotenes. Table 1 also shows that the selection of the proper microbial strains can lead to the production of specific carotenoid types. For instance, carotenoids rich in canthaxanthin can be obtained by the bacterium *Dietzia natronolimnaea* [77]. Apparently, astaxanthin production has not been studied yet implementing cheese whey as substrate. This can be attributed to the fact that *P. rhodozyma* cannot assimilate lactose and galactose [78], whereas among several sugars, lactose results in the lowest astaxanthin production using the microalgae *Chlorella zofingiensis* [79].

Carotenoid production—and particularly the proportions of individual carotenoids—correlate to several factors (e.g., the addition of surfactants and vegetable oils) along with culture conditions, (e.g., aeration rate) [76,80,81,82]. Interestingly, these studies have suggested the potential combination of oil by-products with cheese whey to foster a promising and circular valorization of food by-products for carotenoid generation.

Likewise, pulcherrimin is a red pigment belonging to cyclodipeptides, characterized for its strong biological properties, including antibacterial, antifungal, antitumoral, and anti-inflammatory activities [92]. Türkel et al. [93] mentioned that microorganisms producing pulcherrimin can be effectively used as biocontrol agents against various postharvest pathogens causing fruit and vegetable spoilage, due to the antimicrobial activity of the pigment [93]. Pulcherrimin production has been identified as a metabolite of the yeast *Metschnikowia pulcherrima*, but it has been poorly investigated until now [94,95]. *M. pulcherrima* is able to metabolize various carbon sources, including galactose and glucose, but it cannot hydrolyze lactose [95]. This indicates that hydrolyzed whey lactose could be employed as fermentation feedstock for pulcherrimin production. Alternatively, whey lactose could also be utilized by *Bacillus licheniformis*, which is able to assimilate lactose and has presented the highest pulcherrimin production of 331.7 mg/L under optimized culture conditions [92].

Similarly, flavor and aroma compounds constitute another essential category for the food industry. Those compounds are widely used in order to manufacture attractive products to consumers. Chemical synthesis is an inexpensive method for the production of aroma compounds; however, the derived products cannot be applied in foods. On the other hand, the traditional extraction of aroma compounds from plants exhibits disadvantages regarding low yields and high production cost. In this context, fermentation processes could provide an alternative way for the production of natural aroma compounds. Few studies have focused on cheese whey valorization for the production of fragrances. Several yeast strains were isolated and screened for the production of 2-phenylethanol, an aroma compound found in rose petals, using a whey medium supplemented with sugar beet by-products (molasses, thick juice, or sludge) and L-phenylalanine as a precursor. Among all strains, the highest concentration of 3.3 g/L was achieved by a *Saccharomyces. cerevisiae* yeast strain [96]. The strain *Metschnikowia pulcherrima* is also a promising producer of 2-phenylethanol. Currently, there is not any published study using whey; however, utilization of simulated grape juice medium resulted in significant production of 2-phenylethanol (14 g/L) [97]. Other aroma compounds, including 2-phenylethanol, have been identified at low concentrations in a whey-glucose substrate fermented by the yeast *Wickerhamomyces pijperi.* In total, twelve aroma compounds such as isobutanol, isoamyl alcohol, 2-phenylethanol, acetaldehyde, ethyl acetate, propyl acetate, isobutyl acetate, isoamyl acetate, ethyl butyrate, ethyl propionate, ethyl hexanoate, and ethyl benzoate have been determined [98].

#### 2.2.2. Bacterial Cellulose

Bacterial cellulose is a microbial polysaccharide presenting improved water holding capacity, hydrophilicity, high degree of polymerization, mechanical strength, crystallinity, porosity, and purest fiber network, compared to plant cellulose. Several food applications have been developed for bacterial cellulose, since it has been characterized as a “generally recognized as safe” (GRAS) food by the US Food and Drug Administration (FDA). It has already been applied in ice-creams as a rheology modifier, in confectionery products as a fat replacer, as artificial meat for vegetarian consumers, as a stabilizer of emulsions, or as an immobilization carrier of probiotics and enzymes [99].

Bacterial cellulose is synthesized from several *Acetobacter* species. *Gluconacetobacter xylinus* (formerly known as *Acetobacter xylinum*) is one of the most studied species because of its ability to produce high bacterial cellulose concentrations using various substrates [100]. The implementation of inexpensive renewable resources and agro-industrial wastes as fermentation media could alleviate the high cost for bacterial cellulose production that hinders large scale manufacture. Bacterial cellulose production has been previously studied using by-products deriving from biodiesel and food industries, such as sunflower meal, glycerol, confectionery wastes, citrus by-products, grape pomace, and discarded currants, among others [100,101,102,103,104]. High yields (up to 15.2 g/L) of bacterial cellulose were obtained from synthetic sucrose, glucose, and fructose media [105,106,107]. Agro-industrial substrates such as molasses, fruit juices, or aqueous extracts from citrus residues resulted in production yields of up to 7.8 g/L [101,105]. In the case of lactose utilization, there are only a few studies dealing with bacterial cellulose production (Table 2). Tsouko et al. [100] demonstrated that synthetic lactose was not efficiently metabolized from *Komagataeibacter sucrofermentans* DSM 15973, yielding up to 1.6 g/L bacterial cellulose [100]. Likewise, Mikkelsen et al. [108] reported a final bacterial cellulose production of 0.1 g/L by *G. xylinus* ATCC 53524 grown on galactose [108]. Similarly, other studies have agreed that cheese whey does not support significant bacterial cellulose production by *A. xylinum* 10821, *A. xylinum* 23770 [109], and isolated from Kombucha tea *G. sacchari* [110]. This could be attributed to the fact that the gene that encodes β-galactosidase is not expressed by bacterial cellulose producers. Battad-Bernardo et al. [111] produced a mutant of *A. xylinum* by inserting *lacZ* gene, thus allowing the hydrolysis of lactose [111]. The mutant strain was able to produce 1.82 g/L bacterial cellulose in a whey-based substrate [111]. Another approach lies in the pre-treatment of cheese whey through enzymatic catalysis. In this way, Salari et al. [112] improved bacterial cellulose production (3.55 g/L) by *G. xylinus* PTCC 1734 using an enzymatically-hydrolyzed cheese whey [112]. In another study, around 5.4 g/L of bacterial cellulose was produced by the isolated *Gluconacetobacter. sucrofermentans* B-11267 strain on untreated cheese whey [113].

#### 2.2.3. Functional Food Additives

Development of functional food ingredients is of paramount significance for the food industry, driven by the rising demand to manufacture food products that confer health benefits. Among others, several research studies have been conducted focusing on the production of value-added compounds from mushrooms and microalgae. Still, the full potential of cheese whey was not fully exploited in these cases.

Mushrooms are known for their exceptional functional properties, primarily due to their polysaccharide content [114]. Mushroom fruiting bodies contain a significant amount (35–70%) of non-digestible—and to lesser extent, digestible—carbohydrates. Chitin, β-glucans, glucose, mannitol, and glycogen are the main component carbohydrates [115]. These components serve as the dietary fiber fraction, found mostly in the fungal cell wall, and possess many beneficial effects on human health, including antitumor, hepatoprotective, antimicrobial, prebiotic, antioxidant, hypoglycemic, and hypolipidemic activity [114]. Various mushroom species have been studied for the production of polysaccharides [116,117]. However, the mushrooms belonging to the genus of *Pleurotus* sp., *Ganoderma lucidum,* and *Lentinula edodes* (shiitake mushroom) have been most extensively studied.

Many *Pleurotus* species have demonstrated biological effects, such as immunostimulating and antitumor activity. More specifically, *P. ostreatus* and *P. eryngii* contain several water-soluble and non-soluble β-glucans with prebiotic properties [118]. Recently, Velez et al. [119] showed that the mycelium of *P. djamor* was rich in ergosterol and β-glucans, presenting also high antioxidant activity when grown in cheese whey supplemented with sodium selenite [119]. It was suggested that the lactose-free mycelium, rich in bioactive compounds, is an appropriate supplement for consumers with lactose intolerance. In the case of cheese whey fermentation by *P. sajor-caju*, mycelium biomass was found rich in carbohydrates (arabinose, mannose, and N-acetylglucosamine) and proteins (39.2%) containing high amounts of essential amino acids, such as lysine, leucine, threonine, and phenylalanine [120]. Likewise, *P. osteatus* exhibited higher contents of water-soluble polysaccharides and trace elements, such as calcium, phosphorus, potassium, sodium, and magnesium, when cultivated in whey permeate rather than in synthetic medium [121]. A previous study has shown that cheese whey could also be efficiently utilized in solid state fermentations by *P. ostreatus* [122].

*Lentinula edodes* produces a polysaccharide, namely lentinan, which is the most studied immunomodulating polysaccharide and commercially available in pharmaceutical products. Glycoproteins from this mushroom have been characterized for their antitumor activity [123]. Few reports of *Lentinula edodes* cultivation in cheese whey, have shown that the mycelium is rich in water-soluble polysaccharides and minerals (calcium and potassium) showing also high antioxidant capacity, which suggests that dairy by-products could be utilized as a growth substrate for the cultivation of *L. edodes* [124,125,126].

Few studies have included the optimization of culture conditions using cheese whey for the production of polysaccharides from *Ganoderma lucidum,* indicating its ability to be used as an alternative fermentation medium [127,128,129]. This mushroom has been characterized as “the mushroom of immortality” in Asian countries, because of the existence of 400 different bioactive compounds in mycelia and fruiting body. It is recognized as an alternative adjuvant in the treatment of leukemia, carcinoma, hepatitis, and diabetes, and its bioactive compounds consist of triterpenoids, polysaccharides, nucleotides, sterols, steroids, fatty acids, and proteins, among others [130]. Recently, *G. lucidum* polysaccharides were applied to the production of microcapsules using whey proteins as wall material. This product showed better stability and controlled release ability [131]. This is a representative paradigm of polysaccharide production from medicinal mushrooms, and subsequent product development through encapsulation, in the framework of an integrated cheese whey biorefinery.

Another less studied, yet highly valued, edible mushroom is *Morchella* sp. It is a delicious and expensive mushroom containing heteroglycans with antitumor and hypoglycemic properties [118]. Many species of *Morchella* sp. have demonstrated high polysaccharide content after cultivation on agro-industrial substrates [132]. Nevertheless, the use of cheese whey as substrate for *Morchella* production is still unexplored. Kosaric and Miyata reported, for the first time in 1981, that several *Morchella* species, such as *M. crassipes*, *M. esculenta*, *M. deliciosa*, *M. rotunda,* and *M. angusticeps*, were able to grow on partially deproteinized cheese whey. *M. crassipes* produced the highest biomass of 20 g/L, which contained 45% protein, many essential amino acids, and high proportion of unsaturated fatty acids, such as linoleic acid (55.7%) and oleic acid (13%) [133].

In a similar way, microalgae species are known for their ability to accumulate high quantities of polysaccharides, proteins, polyunsaturated fatty acids, and carotenoids—thus, they are considered superior candidates for food supplements [134]. *Spirulina* and *Chlorella* are blue-green microalgae species exhibiting various health benefits, including antiviral, anti-inflammatory, and antitumor properties [135]. Cultivation of microalgae in cheese whey has been considered a feasible alternative for cost-effective production of microalgal biomass production [136]. More specifically, the mixotrophic culture of *Chlorella vulgaris* in a medium supplemented with hydrolyzed cheese whey demonstrated improved biomass production. This was attributed to the presence of growth-promoting nutrients in cheese whey [136]. *Spirulina platensis* presented higher carbohydrate, carotenoid, and chlorophyll contents when the substrate was supplemented with cheese whey [137,138]. Girard et al. [139] substituted a significant quantity (40%, *v*/*v*) of the basal medium with whey permeate, which resulted in higher biomass production by the fresh water green algae *Scenedesmus obliquus* under mixotrophic conditions [139]. The significance of *Scenedesmus obliquus* is on its enzymatic extracts, which contain several amino acids essential for human diet, presenting also antioxidant and antiviral activities [140,141].

## 3. Whey Proteins: Research Insights and Trends 

**Figure 1:**
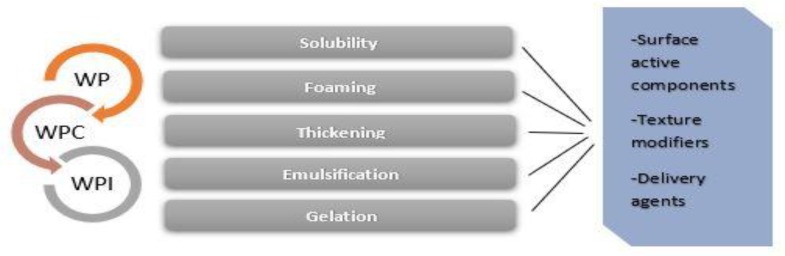
Technological functions of whey protein (WP), whey protein concentrate (WPC), and whey protein isolate (WPI) in food applications.

The increasing global demand for natural ingredients in food manufacturing has led to significant research interest on whey proteins (WP) to manufacture products with desirable characteristics. Whey proteins exhibit physicochemical properties resulting in enhanced texture and quality of end-products, regarding structural and rheological functions [142,143]. Surface-active components, texture modifiers, foaming and gelling agents, thickening agents, emulsifiers, and other bioactivities, among others, indicate targeted application of WP as active ingredients [144,145,146] (Figure 1).

**Figure 2:**
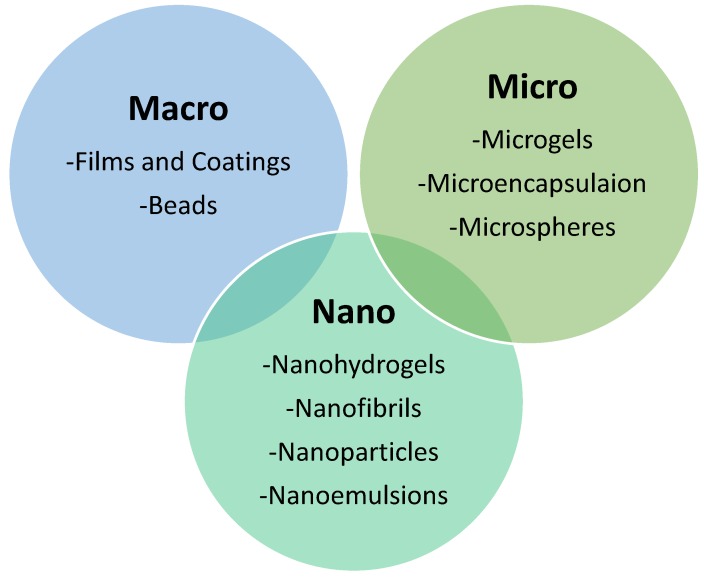
Whey protein systems used as delivery vehicles for bioactive ingredients in food.

WPs can develop macro-, micro-, and nano-structures with numerous promising food applications, such as vehicle carrying for various bio-compounds, flavors, or nutrients (Figure 2). Hence, the emerging development of WP processing techniques could enable further applications that will be directed towards value-added products. In an analogous approach, the following sections will elaborate the most promising applications of cheese whey proteins. The development of edible films and coatings, nanoparticles like hydrogels, and the production of whey protein bioactive peptides as potential nutraceuticals will be further described. Within this context, novel approaches on WP implementation could foster the spectrum of end-use applications that could be incorporated in the development of a holistic process for cheese whey valorization.

### 3.1. Edible Films and Coatings

#### 3.1.1. Recent Strategies for Improved Technical and Functional Properties 

The flourishing demand for eco-friendly active packaging has stimulated research on bio-based packaging. The use of WP for the formation of edible films and coatings has drawn scientific interest, as they are produced from an abundant and renewable material compared to the synthetic counterparts. Among biopolymers used to fabricate edible films, WP exhibits diverse and distinctive technological properties. More specifically, WP can form transparent films and coatings with improved mechanical and barrier properties compared to polysaccharide-based films, indicating them as potential candidate for numerous applications (e.g., high barrier properties like oxygen and volatiles under low moisture conditions) [147]. WPs are usually employed in food application as whey protein concentrates (WPCs) and whey protein isolates (WPIs), induced by the technological advances of whey processing, that convey enhanced functionality (Table 3).

Incorporation of different additive compounds on whey protein-based films to improve their natural, technical and functional properties has lately attracted significant attention [167]. For instance, immunoglobulins (Ig) incorporated into whey protein films improved adhesion and strength [162], but also yielded more transparent and clear films. Moreover, the authors stated that embedding Ig in whey protein matrices resulted in the protection from rapid proteolysis, thereby sustaining their activity in the gastrointestinal track (GIT). Other modifications have been also used to improve whey protein films, including, for instance, the inclusion of lipid components to improve the moisture ability of such films. Almond and walnut oils were employed, along with WPI, leading to a reduction of the surface hydrophilic character of films [148]. Sunflower oil was also used with WPC, resulting in a reduction of water vapor permeability [166]. An emerging strategy to further enhance technical and functional properties of these films, thus improving the compatibility of polymers, lies in the incorporation of nanomaterials [152,156].

Likewise, the use of edible-coated nanosystems has also been considered as a novel approach for food preservation. Findings have lately suggested that novel WPI-based nanocomposites can be part of multilayer flexible packaging films, thus holding great potential to even replace well-established fossil-based packaging materials to support certain mechanical properties during storage [151,153,155,163]. Alternative biomaterials, have also been recently proposed for potential uses in foodstuff applications, via the production of nanocomposites from WPCs activated with lycopene and montmorillonite nanoparticles [164,168]. In another work, WPI nanocomposite films properties were reinforced with oat husk nanocellulose [154]. Evidently, novel perspectives are encountered in the development of novel packaging materials.

Notwithstanding, along with the increase of whey protein edible film generation, it is crucial to overcome specific disadvantages of these films in terms of mechanical features and moisture barrier properties. This could be alleviated by blending films and coatings with various plasticizers. As an example, Basiak et al. (2017) used various starch/whey proteins mixtures and studied their effect on transport properties of the produced films [157].

Likewise, several studies have suggested the use of plasticizers and crosslinking on the formulation of films, as a potential technology for novel food packaging, to improve water resistance, mechanical and barrier characteristics [152], while diversified blends, using different ratios of pullulan or sugars like trehalose, have been also recently proposed as plasticizers of whey protein films [161,169].

#### 3.1.2. Delivery Agents of Bioactive Compounds

The deployment of specific active compounds with antimicrobial or natural antioxidant features, into the matrix of whey protein isolate formation, remains a field of significant importance [170,171]. Improved quality and safety control, along with extended shelf life, of the products, are among the main advantages reported by the use of such complex films, implying their potential application for food wrapping [150,160,172].

Another evolving aspect for bioactive whey protein-based films and coatings was presented via the incorporation of functional bacteria. The use of edible films and coatings as carriers of living microorganisms constitutes a challenge. Microorganisms should remain in high concentrations to exert beneficial effects (antimicrobial or probiotic) without affecting the mechanical or sensory properties of the product. Novel approaches using whey edible films and coatings have resulted in enhanced cell survival. WPC was evaluated, along with several selected biopolymers, as a potential vehicle to investigate *L. rhamnosus* GG survivability [165]. To date, only a few reports exist about probiotic activity in edible films and coatings from whey [164]. The possibility of implementing whey protein formations as a carrier matrix for viable probiotics could potentially result in better survival rates during storage and consumption, thereby promoting novel food applications.

### 3.2. Whey Protein Hydrogels

#### 3.2.1. Formulations and Structural Characteristics

Whey proteins have the ability to form polymeric three-dimensional networks, including hydrogels systems [173,174], and further combine them into nanoparticles. For instance, β-LG, which is the main component of whey protein isolate, is also the responsible particle for the main functional properties. These hydrogels are considered as unique delivery systems, since β-LG nanoparticles are able to bind to hydrophobic compounds. The gel formation process of WP starts when proteins are partially denaturated above critical temperature, leading to the formation of the three-dimensional molecule structure.

Whey protein gels can be temperature-induced by either cold- or heat-set mechanisms [175,176], acid-induced [177], or even enzyme-induced [143,156]. Heat-induced gelation of whey protein is irreversible; therefore, gels are being extensively studied as potential candidates for the preparation of fluid gels without additives [178,179]. Recent studies have reported the preparation of whey protein aggregates through protein crosslinking and building blocks of cold-set gels, thus protecting substances from high temperatures [180,181]. Cold gelation is emerging as a rising method to formulate whey protein microgels [174]. This characteristic renders them as promising candidates to design functional products, where heat- or acid-sensitive nutraceuticals are encapsulated.

Structural characterization of hydrogels is crucial, in order to evaluate their potential in food applications. The rheology of whey protein isolate and casein micelles (MC) mixtures upon heating has been evaluated, indicating that WPI binds to MC and strengthens the junctions of the MC network [176]. Likewise, different mixtures, including starch, rice, or other polysaccharides, or other proteins combined with WPI are currently being studied, aiming to improve the structural and functional properties (e.g., strength, viscosity) of protein solutions [176,182].

The stability and strength of the protein–gel network is evidently associated with the designated applications in food and biomaterials. Characterization of WP gelation profiles has been recently studied; however, the main challenge remains in the production of effective WP microgel systems to overcome brittleness and susceptibility to syneresis [183]. Research is focusing on the ability of WP to form hydrogels that entail specific structural and sensory characteristics for targeted food products like yoghurt, ice cream, bakery products, desserts, and meat products [182]. Understanding the interactions of whey with other biopolymers is crucial in the sense of novel functional food properties. Therefore, the interactions of mixed systems of whey with other biopolymers—such as pectin, κ-carrageenan, xanthan, and basil seed gum—have been studied [184,185,186,187]. These synergistic interactions, leading to the production of stronger gels, could be beneficial in many food formulations such as dairy and dessert products.

#### 3.2.2. Emerging Techniques for Food Applications

Whey proteins have been employed for microencapsulation to enhance the viability of potential probiotic microorganism by using high internal phase emulsions stabilized with WPI microgels [188,189]. Whey protein hydrogels have shown improved survivability of probiotic bacteria under heat treatment at various storage conditions and along GIT passage [190]. Furthermore, numerous kinds of bio-compounds have also been encapsulated, like tryptophan, riboflavin, and peptides from whey protein microbeads [191], vitamins [192], essential oils [193], curcumin [194], lactoferrin [195], and nutraceuticals like folic acid [196]. WPI nanoparticles have been used for α-tocopherol and resveratrol encapsulation as protein-based carriers for hydrophobic components [197]. WPHs have also been successfully applied for encapsulation of water-soluble nutraceuticals [198].

Incorporation of micro- and nanoparticles as carriers of bioactive compounds entails the controlled delivery of these compounds, thus improving nutritional aspects of functional foods. In addition, enhancement of anticancer activity has been also lately reported as a result of controlled release of lycopene loaded in WPI nanoparticles [199]. Actually, various whey-based matrices have been studied in nutritional applications, incorporating different kind of bioactive compounds [191,200], while the use of whey proteins together with fermentable dietary fibers (such as k-carrageenan), has been also recently reported as a suitable vehicle for the inclusion of proteins and peptides in gelled food products [184].

Likewise, production of whey protein nanofibrils, as novel nanocarriers to enhance the solubility of bioactive compounds and control their release into GIT, constitutes an emerging challenge. Within this context, Alavi et al. [201] employed k-carrageenan, in combination with whey protein aggregates (WPA), in order to control the release of curcumin in the upper gastrointestinal tract [201]. In a similar study, Mohammadian et al. [202] observed the high ability of whey protein nanofibrils to bind curcumin, resulting in a significant release in simulated gastric and intestinal fluids [202]. The authors suggested that whey protein nanofibrils could be used in the formulation of food, drinks, and beverages as a multifunctional carrier for bioactive compounds. The produced hydrogels protected curcumin and proved to be effective for colon-specific delivery. Besides the approach of whey protein gel, proteins can independently assemble to form fibrillary systems. These nanosystems convey new insight into food science applications, exhibiting important functional characteristics, including emulsification and gelation properties, increased viscosity competence, and foam stabilization properties at relatively low protein concentration [203,204,205].

On top of that, the fabrication of various types of nutraceutical-carrying nanosystems has recently attracted special interest. Nutraceuticals delivery, to beyond water-soluble compounds, could be achieved by the development of novel whey protein hydrogels. In the frame of that, Hashemi et al. [206] proposed the development of gels, through combination of whey protein solution with nanostructured lipid carriers (NLCs) of fat-soluble compounds [206]. Likewise, whey proteins have been described as effective carriers of lipophilic nutraceuticals and scientific attention has been ascribed to the formation of whey-derived products with inhibitory activity on lipid peroxidation. Zhu et al. [207] used surface hydrophobicity properties to form nanocomplexes consisting of whey proteins and fucoxanthin [207]. Prevention of oxidative degradation of carotenoids or other phenolic compounds has been reported, after the inclusion of the aforementioned compounds into WPI emulsions [208,209,210]. Moreover, increased bioavailability of astaxanthin in Caco-2cell models on whey protein nanodispersion was recently demonstrated [211].

Whey protein nanostructures represent a promising area of food research following their definition as GRAS materials [212]. WPI exhibit core-shell structures similar to natural biopolymers, able to entrap hydrophobic compounds to a great extent. Nanostructural delivery systems are considered substantial approaches to improve biological performance of bioactive compounds. Thus, novel processing techniques are recently employed to modify structural (physicochemicals) and functional properties, demonstrating significant potential for food manufacture applications.

Likewise, pulsed electric field [213], ultrasound [214], or ultraviolet radiation [215] as non-thermal approaches have been shown to exhibit little or no change on the nutritional content. Nevertheless, the electrospinning technique is currently being studied as an emerging advancement for the production of food-grade nanofibers from WP [216]. Electrospinning has been employed to produce micro- to nano-scale fibers as a carrier system to evaluate their potential utilization in food [217]. The ability to generate nanofibers from whey proteins exhibits an opportunity to exploit their inherent benefits, along with the desirable attributes of nanofibers. In addition, incorporation of active compounds and their controlled and monitored subsequent release can be also also achieved [218]. Recent reports dealing with novel electrospun fibers from different blends of WPI have highlighted the advantages of the process, as well as their potential uses in many food related applications [219]. The challenges associated with the development of specific protein fibers correlate with the subsequent specific application, considering that fiber stability in aqueous media and mechanical strength, constitute the most frequent impediments to be challenged [220].

### 3.3. Whey Protein as a Source of Nutraceuticals

#### 3.3.1. Nutritional Aspects of Whey Proteins

Whey proteins are considered functional nutraceuticals, since they exert remarkable biological activities. They mainly consist of lactoglobulin, lactalbumin, bovine serum albumin, lactoperoxidase, lactoferrin, glycomacropeptide (GMP), and immunoglobulins [6]. From a nutritional aspect, whey proteins are superior to other proteins, such as caseins, since their amino acid profile includes a high proportion of essential branched-chain amino acids (BCAAs) [221,222], such as leucine, isoleucine, and valine, which are crucial in blood glucose homeostasis, metabolism, and neural function [223,224,225]. Recently, a Leucyl-Valine peptide in whey protein hydrolysate was found to be able to stimulate heat shock proteins response in rats [226]. Heat shock proteins are known to participate in stabilization and restoration of damaged proteins induced by various stress, resulting in maintaining normal cellular function [227]. Whey proteins also contain significant amount of sulfur amino acids, such as methionine and cysteine, which are reported to act as nutraceuticals [228,229,230]. In general, cheese whey proteins are well-documented as an important source of essential amino acids by means of biologically active peptides. These peptides are considered to be inactive within the sequence of the parent protein, but can be released from whey proteins in sufficient quantities under specific procedures.

Approximately 50% of whey protein is beta-lactoglobulin. Results from a recent study indicate that the Se-β-LG complex presents antitumor activity [231]. The authors also used β- LG nanoparticles as nutraceutical carriers to elevate transepithelial permeation, mucoadhesion, and cellular uptake. In parallel, non-covalent interactions between β-LG and polyphenol extracts of teas, coffee, and cocoa have been reported at pH values of the GIT [232]. The health-promoting effect of cheese whey protein is primarily attributed to their antioxidant properties [233,234] and to their promoting effect on cellular antioxidant pathways [16]. Furthermore, modified products of whey peptides have been shown to increase the antioxidant capacity of the plasma, reducing the risk of certain heart diseases [235]. BSA is another important whey protein, with several drug binding sites, that has been applied as a matrix for nanoparticle-based drug delivery [236].

A broad range of physiological, medical, and nutritional values have been assigned to whey protein and its derivatives, as an excellent source of bioactive peptides. These biomolecules are defined as specific protein fragments that positively influence health and have a beneficial impact on body functions, as summarized in Table 4. Bioactive whey components have been widely studied, revealing various capacities to modulate adiposity, cardiovascular, and gastrointestinal systems [237,238].

#### 3.3.2. Generation of Bioactive Peptides

**Figure 3:**
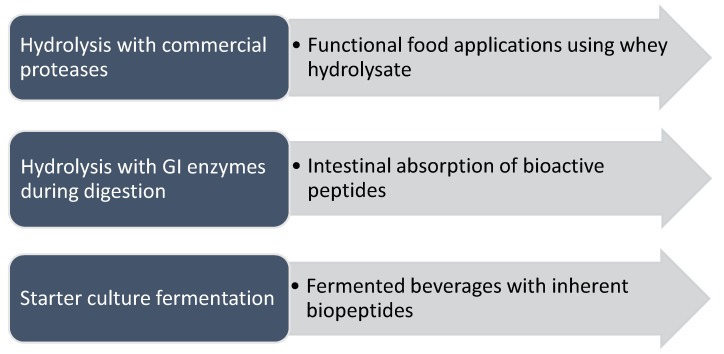
Production methods of bioactive peptides derived from whey proteins and their utilization potential.

Bioactive peptides have attracted significant research and consumer interest, resulting from the potential application in the fortification of products marketed as functional foods or other products for dietary interventions. They can be produced by the following ways: Enzymatic hydrolysis by digestive enzymes during gastrointestinal transit, proteolytic activity of starter cultures during milk fermentation, and proteolytic activity of microorganisms or plants (Figure 3) [257,258,259]. Microbial fermentation remains an easy and cheap way for generating diverse biopeptides through a safe microbial proteolytic food system. Lately, studies have reported that enzymatic hydrolysis of cheese whey proteins results in different biomolecules with antioxidant properties [260], anticancer [261], and even opioid functions [251]. Alvarado et al. [242] studied the production of antihypertensive peptides from whey protein hydrolysate (<3 kDa) and encapsulated them in order to evaluate angiotensin-converting enzyme activity (ACE%) during GIT digestion [242]. The results revealed about a 10% increase of ACE activity by the released peptides.

All three major forms of cheese whey protein and derivatives (concentrates, isolates, and hydrolysates) encompass unique attributes for nutritional, biological, and food ingredient applications. Recent studies have reported that hydrolysates can maximize nutrient delivery to muscle protein anabolism, presenting higher bioactivity [262,263,264]. WPHs have been reported for insulinotropic effects [265]. As stated above, many biopeptides are encrypted within their native protein sequences and can thus be liberated only by protein fragmentation [266].

As far as it concerns the industrial production of whey pure protein fractions, techniques of membrane filtration, such as microfiltration and/or ultrafiltration, are employed to enrich whey food ingredients (i.e., whey hydrolysates) [267,268]. Herein, the challenge remains in terms of stability of these fractions under different downstream processes and gastrointestinal phases.

Regardless the scientific achievements, application of bioactive peptides from whey that exert beneficial effect in human nutrition is in its infancy, as several challenges exist in the discovery and identification of bioactive peptide both in vitro and in vivo [269]. Novel bioprocessing strategies for bioactive peptides have been developed as food peptidomics, food proteomics, and nutrigenomic [270,271]. Evidently, an omics approach and bioinformatics could elucidate further development on the application of bioactive peptides [272].

The versatile end applications of whey proteins render them compounds of paramount importance, to be further employed within the frame of integrated biorefineries concepts. However, further research is required to elucidate the underlying mechanisms on food fortification and in human nutrition. Nonetheless, sedimentation and recovery of protein fraction, separation of individual whey proteins and derivatives synthesis have great potential to be incorporated in an integrated process and further enhance feasibility by obtaining end-products of high-value.

## 4. Current Integrated Biorefineries

As per the IEA Bioenergy Task 42 definition, “Biorefinery is the sustainable processing of biomass into a spectrum of marketable products (food, feed, materials, chemicals) and energy (fuels, power, heat)” [273]. Thus, to ensure the sustainability and cost-effectiveness of a biorefinery, it is indisputable that a multitude of viable end-products should be manufactured. The development of a biorefinery concept should also employ all potential streams of the onset feedstock under the concept of circular economy and zero waste generation, thus encompassing all three pillars of sustainability, i.e., environment, society, and economy [273]. Integration of biorefining processes annexed to existing manufacturing plants for on-site valorization would alleviate the industry and stake holders’ concerns about investing in facilities and equipment that would be depreciated. Another key parameter during the configuration of biorefinery concepts is the complex and heterogenous nature of renewable resources, particularly food waste. The complexity of food waste by-products also impairs the economic assessment of such biorefineries. In any case, high value-added bio-based products deriving from food waste and agricultural by-products should be generated in large amounts, whereas the final market price should be competitive with the chemically produced counterparts [274].

The co-production of fuels and platform chemicals has been the main driver for the configuration of biorefinery scenarios. Biorefining of food waste implements bioprocesses like acidogenesis, fermentation, solventogenesis, oleaginous processes, etc., that yield several products like biofertilizers, animal feed, and biochemicals [14,275]. Nonetheless, bioenergy production is a constructive process for biomass; therefore, to maintain the sustainability, it is advocated that a wide range of bio-products are obtained, instead of a single line product. Several researchers have highlighted the importance of recycling agricultural and industrial wastes through biotransformation by applying a biorefinery concept, utilizing waste as the main feedstock [276].

As previously stated, cheese whey is found among the most significant (and unavoidable) industrial waste streams. Proper disposal and reuse strategies, through bioprocess integration, are unequivocal to mitigate the vast amounts of non-avoidable food waste. Currently, conventional treatments for cheese whey include landfill disposal or anaerobic digestion with focus on BOD and COD reduction, rather than the production of biochemicals, bioenergy, and other value-added novel products [277]. Thus, new approaches for refining have been applied to convert dairy by-products into several valuable bio-based products, such as feed additives, bioplastics, and biochemicals—but also generate high-volume yet lower-value products (bioethanol), or high-value but low-volume products, as nutraceuticals [278].

As it was reported in the previous sections, approaches for cheese whey valorization so far do not exploit the full potential, particularly with respect to food applications. From one point, the protein fraction of cheese whey has been employed to obtain WP to manufacture edible films and coatings, hydrogels, and nutraceuticals (bioactive peptides), among others. The enhanced nutritional value, along with the health benefits, that whey proteins confer [238] have established whey as a high-value raw material in the food industry, with emerging and novel applications. On the other hand, lactose is a precursor substrate for the fermentative synthesis of lactic acid, succinic acid, and polyhydroxyalkanoates, among others [14]. Lactose can be directly fermented by microorganisms, e.g., *Lactobacillus casei*, *Lactobacillus acidophilus*, *Lactobacillus delbrueckii*, *Lactobacillus plantarum,* and *Lactobacillus rhamnosus* [27,279,280,281,282,283]. For instance, lactic acid is an organic biodegradable acid, extensively used in the pharmaceutical, textile, and food industries, where it has been employed in polymerization reactions to produce polylactic acid as a biodegradable polymer. Moreover, lactose deriving from cheese whey can provide a nutrient feedstock for the fermentative production of polyhydroxyalkanoates [284], demonstrating specific physical and mechanical properties.

Biorefining has been already applied for the bioconversion of lactose from cheese whey into several valuable bio-products [10,285,286,287]; still, the majority of previously reported studies do not implement the complete capacity of cheese whey for food-based formulations. However, within the concept of integrated and consolidated bioprocesses, all streams should be exploited to yield multiple end-products. Likewise, by-products from whey protein manufacture are whey permeate and, following the extraction of lactose, delactosed whey permeate. These dairy-processing side-streams lack effective disposal or further exploitation routes; thus, they can be implemented to formulate cost competitive bio-based products under the concept of sustainable bio-economy targeting “zero waste” [288,289]. Equally, another possible alternative would be to combine different side streams, deriving from separate food processing industries, to configure bioprocesses for multiple end-products.

For instance, a recent study reported the fermentative production of bacterial cellulose (BC) using side-streams of Corinthian currants finishing (CFS), with high antioxidants and sugar content, via the evaluation of nitrogen sources addition and cheese whey [290]. Response surface methodology was applied to evaluate the conditions for BC production for CFS/cheese whey mixtures, concluding that optimum results were achieved on 50.4% whey permeate and 1.7% yeast, at pH 6.36. On top of that, texture analysis was also conducted, indicating that BC could be implemented to formulate foods with potential prebiotic effects, thereby enhancing the functionality of the end-product. Within the same concept, wine lees were combined with cheese whey to develop a biorefining process with microbial oil as the target product [291]. The principal element to initiate process design was the utilization of carbon source from cheese whey (lactose) and the nitrogen source from wine lees, to substitute conventional and expensive chemicals. Polyphenol-rich extracts and tartaric salts, along with crude enzymes via solid state fermentation, were obtained via the treatment of wine lees to yield a nutrient-rich fermentation feedstock. Whey protein concentrate (WPC) was generated after membrane filtration to recover lactose, and at the end of the fermentation process, yeast cell mass could be used as animal feed. The proposed process induced the production of several end-products that could find diversified applications based on market demand, particularly in food formulation (antioxidants, microbial oil, whey protein). It is also worth noting that all streams were exploited, leading to minimal waste generation and enhanced economic feasibility.

A dairy waste biorefinery considering the treatment of cheese whey and cattle manure was proposed and presented by Chandra et al. [28]. Briefly, manure was directed to anaerobic digestion to yield volatile fatty acids, biomethane, hydrogen and fertilizers, considering that biofuel production is often included during a biorefinery design. On the other hand, cheese whey was employed in a more complete valorization scheme to obtain various products. In line with this, lactose was either used for GOS synthesis or for the fermentative production of lactic acid. Alternatively, an enzymatic hydrolysis step for lactose was suggested, prior to alcoholic fermentation or anaerobic digestion, to formulate ethanol. The proposed bioprocess entailed increased viability and sustainability, suggesting also almost zero waste; however, it could be further developed to a configuration that would separately exploit whey protein and lactose into more targeted high value-added food applications, as will be discussed in the following section.

Within the concept of targeted and novel food applications, whey was employed to produce a novel dried cheese whey using thermally-dried *L*. *casei* ATCC 393 and *L*. *delbrueckii* ssp. *bulgaricus* ATCC 11842, which were immobilized on casein [292]. Sensory analysis was also performed, and the results clearly demonstrated a novel probiotic product with enhanced aroma, improved shelf life, and protection from pathogenic strains. Hence, to induce novel value-added compounds, it is vital to develop a cheese whey valorization process that is directed principally on products that can separately stand as functional foods or components that are used in food formulation with enhanced properties.

## 5. Innovative Refining Processes of Cheese Whey and Future Perspectives in Food Applications

Cheese whey is an abundant and low-cost renewable resource deriving from the cheese industry. Utilization of food-grade enzymes and microbial cells, or chemical modification, indicate economically viable approaches for the conversion of cheese whey to produce functional food products. The growing worldwide demand for added-value food products, exhibiting functional properties, is accompanied by an equally increasing market of the latter products to meet consumers’ demands. Meanwhile, the transition to bio-economy era demands the sustainable production of these foods, thereby impeding the development of effective bioprocessing and integrated strategies.

**Figure 4:**
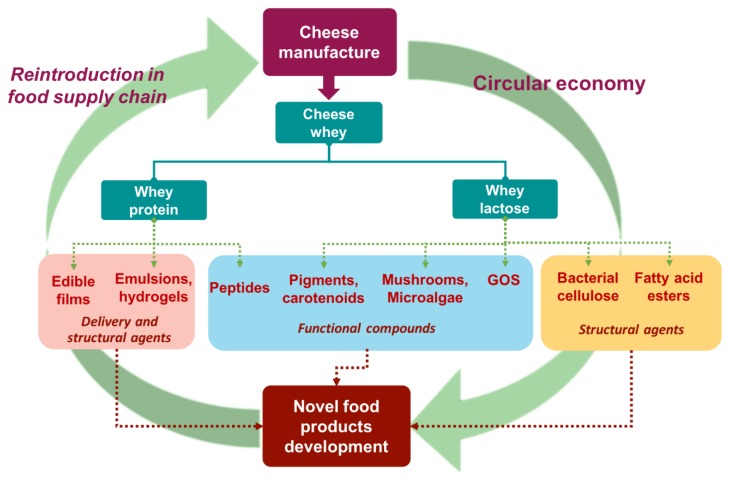
Proposed cheese whey-integrated biorefineries targeting food applications within the circular economy context.

The current article demonstrates state-of-the-art processes for cheese whey valorization, towards the manufacture of emerging food additives and products. The majority of the configured processes are focused either only in protein or lactose streams from cheese whey. Evidently, published studies on integrated biorefineries using cheese whey are inadequate with respect to the investigation of both lactose and protein streams to yield food-based compounds. In the era of circular economy, the implementation of several processing methods should be indispensably included within a consolidated cheese whey biorefinery to generate functional foods with improved properties (Figure 4). The following suggested examples relating to food applications indicate solid perspective of novel integrated biorefinery approaches for high value-added food production.

An interesting paradigm to be potentially integrated for cheese whey valorization was introduced by Paximada et al. [293], where whey protein emulsions using bacterial cellulose were produced as an alternative to commercial thickeners, such as xanthan gum and locust bean gum [293]. Xanthan gum is considered an important food thickener, providing high shear-thinning behavior in food products [294]. Worth noting is that Paximada et al. [293] stated that bacterial cellulose had a better shear thinning profile than xanthan gum [293]. Results indicated that lower bacterial cellulose concentration was required compared to xanthan gum or locust bean gum to obtain emulsions with similar rheological properties, and also that bacterial cellulose produced from food by-products is a cheaper alternative to commercial gums [293]. The interaction between whey protein and bacterial cellulose was investigated by Peng et al. [295], noting that bacterial cellulose modified the properties of whey protein fibrillar gel [295]. Particularly, bacterial cellulose addition resulted in a bifibrillar gel, improving whey protein fibril alignment rather than the absence of bacterial cellulose [295].

In Section 2.2.3, we demonstrated that the fermentation of *Spirulina* in cheese whey is an unexploited bioprocess, regardless of the several attempts performed to incorporate the highly nutritious *Spirulina* in foods [134]. Particularly, *Spirulina* has been used as a healthy additive for novel ice cream production with high nutritional value [296]. The estimated cost of the final product was found to be cost competitive within the functional products segment [296]. The addition of *Spirulina platensis* in soft cheese significantly enriched the protein and carotenoid content of the final product [297]. Furthermore, the protein concentrate extracted from the biomass of *Spirulina* sp. has been utilized as functional coating material to encapsulate pigments of commercial interest, such as phycocyanin. Although *Spirulina* sp. is a rich source of this pigment, the production of ultrafine fibers by the electrospinning method resulted in increased thermal stability of phycocyanin [298]. *Spirulina* sp. and *Chlorella* sp. have also been employed for the production of many food products, such as gels [299,300] and fermented milk [135]. In the production of functional fermented milk products, the co-addition of these microalgae and probiotics increased the viability of the probiotic bacteria [135]. Additionally, Terpou et al. [301] reported that whey protein hydrolysate and whey protein concentrate can also promote probiotic viability [301]. These studies indicate that the utilization of both whey protein products and microalgae, previously grown on whey lactose, could be employed for the development of probiotic products with enhanced beneficial value.

This article showed that several protein sources, including *Spirulina* and whey protein, could act as delivery agents for bioactive compounds, including pigments, carotenoids, or GOS, which are produced via microbial or enzymatic bioprocesses using lactose from whey. Additionally, compounds such as lactose esters and bacterial cellulose can be utilized as thickeners and emulsifiers, altering the rheological behavior of food [66,293].

Therefore, to conform to the concept of circular economy that would ideally allow the reintroduction of produced bio-based food components in the food chain, there is a necessity to undertake novel approaches for biorefinery development, considering also the founding pillars of economy, society, and environment. In a complete cascading process, cheese whey would be initially treated to obtain the protein rich fraction and whey lactose. Whey protein fraction could be treated to result in the formulation of nutrient supplements, encapsulation agents of the fermentative production of *Spirulina,* and further inclusion in food. Equally, whey protein could be employed to encapsulate probiotic strains, to manufacture end-products with enhanced nutritional value and sensory characteristics. On the other hand, the lactose-rich stream could be efficiently valorized via enzymatic and microbial bioconversion processes. Synthesis of GOS, either with crude or commercial enzymes, confers a notable option, considering the prominent increase in the market of prebiotics. Purified GOS can be obtained via conventional methods (activated charcoal, membranes, etc.) or via the use of yeast strains (*Kluyveromyces marxianus*, *Saccharomyces cerevisiae*) that consume the unreacted lactose, glucose, and galactose. Appropriate selection of strains in the latter case could lead to additional value-added product formation, whereas the yeast cells after GOS recovery could serve as potential animal feed supplements. The suggested scenario resembles the dairy waste biorefinery proposed by Chandra et al. [28]; however, we elaborate more on high value-added products that will find end applications in the food industry rather than biofuels [28].

Within this concept, our research group at the Department of Food Science & Technology of the Ionian University is currently focusing on the development of an integrated cheese whey biorefinery scheme. More specifically, lactose deriving from whey will be used for the production of potential probiotic starter cultures from non-dairy *Lactobacillus* strains and the production of bacterial cellulose. Subsequently, bacterial cellulose will be combined with whey protein to form immobilization support matrices for probiotic cultures that will be further incorporated in dairy products (e.g., cheese, yogurt). Overall, it is envisaged to configure a refining process to implement both lactose and protein streams, resulting in high value-added products that will be introduced in the food manufacturing sector (Figure 4). It is easily deduced from these studies that intensive and contemplated effort is conducted to establish an integral process to appraise a closed-loop food supply chain through the manufacture of novel food products. Ultimately, novel approaches will yield alternative bio-based components exhibiting enhanced physicochemical properties, sensory characteristics, and nutritional value.

## Figures and Tables

**Table 1:** Carotenoid production from various microorganisms through fermentation in cheese whey (CW).

Microorganism	Supplementation of CW Medium	Composition of Total Carotenoids	Concentration (mg/L)	Yield (mg/g) ^1^	Reference
*Blakeslea trispora* ATCC 14271 & ATCC 14272	Tween 80, Span 80, β-ionone	β-carotene, γ-carotene, lycopene	1620.0	222.0	[76]
*Blakeslea trispora* ATCC 14271 & ATCC 14272	Tween 80, Span 80, β-ionone	β-carotene, γ-carotene, lycopene	1360.0	175.0	[83]
*Blakeslea trispora* ATCC 14271 & ATCC 14272	Tween 80, Span 80, vegetable oils	β-carotene, γ-carotene, lycopene	~672.0	16.0	[82]
*Blakeslea trispora* ATCC 14271 & ATCC 14272	Tween 80, Span 80, vegetable oils, antioxidants and other nutrients	β-carotene	350.0	11.6	[84]
*Blakeslea trispora* ATCC 14271 &ATCC 14272	Tween 80, Span 80, vegetable oils	N.S. ^2^	376.0	8.0	[85]
*Mucor azygosporus* MTCC 414	Soluble starch	β-carotene	3.5	0.38	[86]
*Rhodotorula mucilaginosa* NRRL 2502		N.S. ^2^	70.0	29.2	[80]
*Rhodotorula mucilaginosa* CCY 20-7-31		β-carotene	11.3	0.38	[87]
*Rhodotorula glutinis* CCY 20-2-26		β-carotene	51.2	1.48	[87]
*Rhodotorula rubra* GED5 co-culture with *Kluyveromyces lactis* MP11		Torularhodin, β-carotene, torulene	10.2	0.42	[88]
*Rhodotorula glutinis* 22P co-culture with *Lactobacillus helveticus*		β-carotene, torularhodin, torulene	8.09	0.27	[89]
*Rhodotorula rubra* GED8 co-culture with *Lactobacillus bulbaricus, Streptococcus thermophilus*		β-carotene, torulene, torularhodin	13.1	0.50	[90]
*Sporidiobolus salmonicor* CBS 2636		N.S. ^2^	0.91	0.25	[91]
*Sporobolomyces roseus* CCY 19-4-8		β-carotene	29.4	2.89	[87]
*Dietzia natronolimnaea* HS-1		canthaxanthin (2.87 mg/L)	3.06	~0.9	[77]

^1^ Yield expressed as milligrams of carotenoids per gram of dried biomass; ^2^ N.S.: Not specified.

**Table 2:** Bacterial cellulose (BC) production using lactose or lactose derivatives.

Microorganism	Carbon Source	BC (g/L)	Reference
*K. sucrofermentans* DSM 15973	Synthetic lactose	1.6	[100]
*G. xylinus* ATCC 53524	Synthetic galactose	0.1	[108]
*A. xylinum* 10821	Cheese whey	0.04	[109]
*A. xylinum* 23770	Cheese whey	1.13	[109]
*G. sacchari*	Cheese whey	0.15	[110]
*A. xylinum* mutant	Cheese whey	1.82	[111]
*G. xylinus* PTCC 1734	Hydrolyzed cheese whey	3.55	[112]
*G. sucrofermentans* B-11267	Cheese whey	5.4	[113]

**Table 3:** Edible films formation from whey protein isolate (WPI) and whey protein concentrate (WPC) and their functional features.

Substrate	Promoting Compound	Functionality	Reference
**WPI**	Almonds, walnut oil	Water barrier improvement	[148]
	β-cyclodextrin/eugenol, carvacrol	Antimicrobial component delivery	[149]
	Lysozyme	Antimicrobial component delivery	[150]
	Montmorillonite nanoplatelets	Oxygen barriers improvement	[151]
	Montmorillonite clay nanoparticles	Thermal stability, water vapor permeability	[152]
	Nanocrystalline cellulose, transglutaminase	Improved mechanical properties	[153]
	Oat husk nanocellulose	Enhanced tensile strength, solubility, decreased elongation at break and moisture content, decreased transparency and water vapor permeability	[154]
	Pullulan, montmorillonite	Improve the mechanical properties, thermal properties, and water resistance	[155]
	Sodium laurate-modified TiO_2_ nanoparticles	Water vapor permeability decreased, tensile strength increase, decreased transparency	[156]
	Starch	Water vapor permeability, microstructure	[157]
	Zein	Enhanced water solubility and heat-sealablity	[158]
	Zein nanoparticles	Improved moisture barrier and mechanical properties	[159]
**WPC**	Cinnamon essential oil	Antimicrobial	[160]
	Glucerol, pullulan, beeswax	Improved color indices, diminished water solubility and water vapor permeability, and increased tensile strength	[161]
	Immunoglobulins	Increase stickiness, adhesion, and tensile strength of the films	[162]
	Liquid smoke	Antimicrobial/improved mechanical properties	[163]
	Montmorilonite, lycopene	Antioxidant activity and UV-vis light protection/mechanical properties improvement	[164]
	Rosmarinic acid, carnosol, carnosic acid		
	Sodium alginate, pectin, carrageenan, locust been gum/*L. rhamnosus*	Enhanced survival during drying and storage, reduced film water vapor permeability	[165]
	Sunflower, beeswax	Water vapor permeability	[166]

**Table 4:** Whey protein edible film formation from whey protein isolate (WPI) and whey protein isolate (WPI) and their functional features.

Biological Function	Formulation	Test Model	References
Anti-diabetic	Whey protein hydrolysate	Insulin-resistant rats	[239]
	Whey protein	Human	[240]
Anti-inflammatory	β-lactoglobulin hydrolysate	In vitro	[241]
Anti-hypertensive	Whey protein concentrate	In vitro	[242]
Anti-obesity	Whey protein concentrate	Obese human	[243]
	Whey protein concentrate	Obese human	[244]
Antitumor	β-lactoglobulin hydrolysate	*In vitro*	[231]
Benefit in resistant exercise	Hydrolyzed whey protein	Human	[245]
Blood pressure lowering	Whey protein hydrolysate	Rats	[246]
Dermatoprotective	Whey peptide	Mice	[247]
GI motility	Whey protein concentrate, Whey protein Hydrolysate	Mice	[248]
Gut and energy homeostasis	Whey protein isolate	Mice	[249]
Hypolipidemic	Whey protein	Mice	[234]
Muscle protein synthesis/glycogen content	Whey protein hydrolysate	Mice	[226]
Osteroprotection	Whey protein derived dipeptide Glu-Glu	In vitro	[250]
Oxidative stress	Whey protein concentrate	Mice	[251]
Oxidative stress/Glucose metabolism	Whey protein isolate	Overweight/obese patients	[252]
Phenylketonouria therapy	Whey protein glycomacropeptide	Human/mice	[253]
Recovery of muscle functions	Whey protein hydrolysate	Human	[254]
	Whey protein	Human	[255]
Sceletical muscle protection	Whey protein hydrolysate	Rats	[256]
